# Expression and phylogenetic analyses reveal paralogous lineages of putatively classical and non-classical MHC-I genes in three sparrow species (*Passer*)

**DOI:** 10.1186/s12862-017-0970-7

**Published:** 2017-06-26

**Authors:** Anna Drews, Maria Strandh, Lars Råberg, Helena Westerdahl

**Affiliations:** Department of Biology, Lund University, Ecology Building, 223 62 Lund, Sweden

**Keywords:** MHC class I, Passer, sparrows, Classical genes, Non-classical genes, gene expression

## Abstract

**Background:**

The Major Histocompatibility Complex (MHC) plays a central role in immunity and has been given considerable attention by evolutionary ecologists due to its associations with fitness-related traits. Songbirds have unusually high numbers of MHC class I (MHC-I) genes, but it is not known whether all are expressed and equally important for immune function. Classical MHC-I genes are highly expressed, polymorphic and present peptides to T-cells whereas non-classical MHC-I genes have lower expression, are more monomorphic and do not present peptides to T-cells. To get a better understanding of the highly duplicated MHC genes in songbirds, we studied gene expression in a phylogenetic framework in three species of sparrows (house sparrow, tree sparrow and Spanish sparrow), using high-throughput sequencing. We hypothesize that sparrows could have classical and non-classical genes, as previously indicated though never tested using gene expression.

**Results:**

The phylogenetic analyses reveal two distinct types of MHC-I alleles among the three sparrow species, one with high and one with low level of polymorphism, thus resembling classical and non-classical genes, respectively. All individuals had both types of alleles, but there was copy number variation both within and among the sparrow species. However, the number of highly polymorphic alleles that were expressed did not vary between species, suggesting that the structural genomic variation is counterbalanced by conserved gene expression. Overall, 50% of the MHC-I alleles were expressed in sparrows. Expression of the highly polymorphic alleles was very variable, whereas the alleles with low polymorphism had uniformly low expression. Interestingly, within an individual only one or two alleles from the polymorphic genes were highly expressed, indicating that only a single copy of these is highly expressed.

**Conclusions:**

Taken together, the phylogenetic reconstruction and the analyses of expression suggest that sparrows have both classical and non-classical MHC-I genes, and that the evolutionary origin of these genes predate the split of the three investigated sparrow species 7 million years ago. Because only the classical MHC-I genes are involved in antigen presentation, the function of different MHC-I genes should be considered in future ecological and evolutionary studies of MHC-I in sparrows and other songbirds.

**Electronic supplementary material:**

The online version of this article (doi:10.1186/s12862-017-0970-7) contains supplementary material, which is available to authorized users.

## Background

The major histocompatibility complex (MHC) is a key component of adaptive immunity and holds the most polymorphic genes known in the vertebrate genome [1]. MHC class I (MHC-I) proteins are expressed on all nucleated cells whereas MHC-II proteins are expressed only on antigen presenting cells [2]. Typically, animals have a handful of functional MHC-I genes, as exemplified by humans (six genes), swine (*Sus scrofa domesticus*; six genes) and domestic chicken (*Gallus gallus*; four genes) [3–5]. On the contrary, songbirds of the order Passeriformes have a larger number of MHC-I genes than most other species investigated to date [6–8]. O’Connor et al. (2015) reported between four and 20 MHC-I genes per individual across Passerida (i.e. genomic MHC-I exon 3 sequences in open reading frame), and Biedrzycka et al. (2017) found 65 alleles per individual, i.e. at least 33 MHC-I genes, in the sedge warbler *Acrocephalus schoenobaenus* [8, 9]. The functional significance of all these MHC-I gene copies in songbirds is not known.

In most species studied to date — for example humans and other primates, swine, mice and chicken — MHC-I genes are categorized as classical or non-classical MHC-I genes [3, 5, 10, 11]. In mammals, classical MHC-I genes (MHC-Ia) are highly polymorphic and highly expressed, whereas non-classical (MHC-Ib) are less polymorphic and have low expression [12, 13]. The non-classical genes do not appear to have a common origin among distantly related mammals but seem to have arisen independently from recent duplications of classical MHC-I genes within species [14]. MHC-Ia molecules play an important role in adaptive immunity by presenting peptides to T-cells [1], whereas MHC-Ib molecules have other immune functions [12, 13, 15, 16]. Humans have three MHC-Ia genes (HLA-A, -B and -C) and three MHC-Ib genes (HLA-E, -F and -G) [3]. HLA-A, -B and -C have a much larger number of alleles (>9000 world-wide) than HLA-E,-F and -G (<90 world-wide) [17]. HLA-A, -B and -C genes are expressed in most tissues, but there are gene-specific expression differences among the genes; HLA-C is expressed to a lower degree than HLA-A and -B resulting in high variation in expression levels among classical genes [18].

Classical and non-classical class I genes have also been reported in birds of the order Galliformes, e.g. in chicken, turkey (*Meleagris gallopavo*) and golden pheasant (*Chrysolophus pictus*) and here the MHC-Ia and MHC-Ib genes are referred to as MHC-B and MHC-Y, respectively [4, 19–21]. The chicken has two classical MHC-I genes at the MHC-B locus and two non-classical MHC-I genes at the MHC-Y locus [4]. Both genes at the classical B-locus are expressed, but the ‘major’ gene is highly expressed compared to the ‘minor’ gene [22–24]. Only one of the non-classical Y-locus genes has been shown to be expressed and then specifically in spleen [25, 26]. The presence of classical and non-classical MHC-I genes is less established in non-galliform birds, but has been suggested in species of the orders Anseriformes, Charadriiformes and Pelecaniformes [27–30]. Moreover, in Anseriformes and Pelecaniformes there seem to be one putatively classical gene that is highly expressed [30, 31].

MHC genes evolve by frequent gene duplications, but there is also gene loss when gene copies become non-functional, and the evolution of MHC therefore fit a birth-and-death model of molecular evolution [32–34]. The large number of MHC-I genes in the genomes of songbirds indicates either a higher rate of gene duplications in songbirds compared to other animals, or that several copies of their MHC genes have been duplicated simultaneously [6–8]. Very little is known about neo-functionalization among the multiple gene copies in songbirds, though it seems likely that some gene copies have evolved different functions, like the classical and non-classical genes found in other species [27–30]. It is important to distinguish non-classical and classical MHC genes in evolutionary and ecological studies since only the latter are subject to balancing selection and expected to be associated with disease resistance and fitness.

Karlsson and Westerdahl [35] showed that house sparrows (*Passer domesticus*) have two types of MHC-I alleles that exhibit some of the hallmarks of classical and non-classical genes. The putatively non-classical house sparrow MHC-I alleles have low levels of polymorphism, few positively selected sites and form a distinct phylogenetic cluster, whereas the putatively classical MHC-I alleles have high polymorphism, many positively selected sites and do not form a supported phylogenetic cluster [35]. The putatively non-classical alleles in house sparrows are easily identified by a six base pair deletion in exon 3 [35, 36]. However, the expression pattern of these putatively classical and non-classical genes in house sparrow has not been investigated. Hence, a way to further establish the occurrence of classical and non-classical MHC genes in house sparrows would be to measure their relative expression. Classical genes are often highly expressed compared to non-classical genes, and certain genes within each category might be more highly expressed, e.g. if ‘major’ and ‘minor’ loci are present as indicated in species of the bird orders Galliformes, Anseriformes and Pelecaniformes [25–30].

Previous studies on the evolution of MHC genes have shown that orthologous classical MHC genes often survive longer in the genome—over speciation events—than non-classical genes [10]. A next step to continue studying putatively non-classical genes in Passerines is therefore to investigate their occurrence in species that are closely related to house sparrows. Finding putatively classical and non-classical genes in several species would not only make the finding more solid but also give an indication of their evolutionary age.

We set out to investigate the structure, number and expression patterns of MHC-I genes among three sparrow species, the house sparrow, the Spanish sparrow (*Passer hispaniolensis*) and the tree sparrow (*Passer montanus*), in order to identify neo-functionalization of MHC-I genes, in particular whether there are both putatively classical and non-classical genes. We firstly reconstruct allelic phylogenies where we classify the sparrow MHC-I alleles as putatively classical or non-classical genes and then test if these putatively classical and non-classical alleles differ in i) number of genomic alleles between species, ii) number of expressed alleles between species and iii) relative gene expression within species.

## Methods

### Study species

We study three species of sparrows in the Passer clade; house sparrow (*Passer domesticus*) (*n* = 5), Spanish sparrow (*P. hispaniolensis*) (*n* = 3) and tree sparrow (*P. montanus*) (*n* = 5)*.* The native range of the house sparrow and tree sparrow covers most of Eurasia whereas the Spanish sparrow has a more restricted distribution around the Mediterranean Sea and in south-west Asia [37]. All three species live in both urban and rural environments [37]. In order to determine when house sparrow, Spanish sparrow and tree sparrow separated phylogenetically a maximum clade credibility tree was constructed based on data from 23 Passer species and an outgroup (*Cyanistes caeruleus*) from the Bird Tree website [38, 39], for details see Additional file 1: Method S1. House sparrows and Spanish sparrows split 3 million years ago, while the tree sparrows are more distantly related and split from the other two species 7 million years ago.

### Sample collection and extractions

House sparrows and tree sparrows were caught, with mist nets, in Löberöd, Skåne, Sweden. The Spanish sparrows were kept and caught in aviaries at University of Oslo, Norway. All samples were collected during the autumn of 2012. Blood samples (20-40 μl) were taken from the brachial vein and then stored either at −20 °C in SET buffer (150 mM NaCl, 50 mM TRIS, 1 mM EDTA, pH 8.0), for DNA extraction, or at 4 °C in 100 μl K_2_EDTA and 500 μl TRIzol LS (Life Technologies, Carlsbad, CA, USA), for RNA extraction. DNA was extracted with ammonium acetate extraction [40]. RNA was extracted with a combination of the TRIzol LS protocol (Life Technologies, Carlsbad, CA, USA) and the RNeasy Mini kit (QIAGEN, Hilden, Germany). Briefly, the homogenization and phase separation was done according to the TRIzol LS protocol, resulting in an aqueous phase. One volume of 70% EtOH was added to the aqueous phase and from this step the RNeasy protocol was followed, including an on-column DNase treatment [41]. The RNA (mRNA) was reverse transcribed to complementary DNA (cDNA) using the RETROscript kit (Life Technologies, Carlsbad, CA, USA) according to the manufacturer’s protocol.

### High-throughput amplicon sequencing

We sequenced partial MHC-I exon 3 amplicons (185–226 bp) obtained from genomic DNA (gDNA) and cDNA (to examine gene expression) from house sparrows, tree sparrows and Spanish sparrows using 454 amplicon sequencing. Four different primer combinations were used to amplify MHC-I exon 3 alleles in each species to minimize the effects of amplification bias and allelic dropouts which is often a problem when using only one primer pair (Additional file 1: Table S1, Figure S1). Primer combinations 1 and 2 amplify both putatively classical and non-classical alleles whereas primer combination 3 and 4 exclusively amplifies putatively classical and non-classical alleles, respectively [8, 35, 42]. Each individual was represented by one gDNA and one cDNA sample and samples were technically duplicated (two PCRs from 40% of the samples). We performed PCR with individually tagged 454 fusion primers (6-bp tag on forward and reverse primer) [43]. Each 15 μl PCR reaction contained either 25 ng gDNA or 10 ng cDNA, 0.2 μM of each primer and 1× QIAGEN Multiplex PCR Master Mix (QIAGEN, Hilden, Germany). The cycling conditions for primer combination 1 and 2 were set to 35 cycles at 95 °C (30s), 60 °C (90s), 72 °C (60s) followed by 72 °C for 10 min. For primer combination 3 and 4 the cycling conditions were 30 cycles at 95 °C (30s), 65 °C (60s), 72 °C (60s) followed by 72 °C for 10 min. The PCR products were verified on a 2% agarose gel and products were pooled semi-equimolarly based on the strength of the bands with a maximum of eight products per pool. These pools were purified on MinElute PCR purification columns (QIAGEN, Hilden, Germany) according to the manufacturer’s protocol and quantified on a NanoDrop 2000/2000c (Thermo Fisher Scientific, Wilmington, DE, USA). The purified pools were pooled in equimolar DNA amounts in one final pool per primer combination. Amplicons were sequenced in two separate 454 sequencing runs (one for primer combination 1 and 2 amplicons and another for primer combination 3 and 4 amplicons) at the Lund University DNA Sequencing facility, Faculty of Science, Sweden.

### Filtering of high-throughput sequencing data

There are errors associated with high-throughput sequencing techniques that will result in artefactual alleles (AA). The AAs were distinguished from the true alleles (TA) and removed from the dataset by a number of filtering steps, (for details see Additional file 1: Methods S2). Briefly, the first filtering step handled AA originating from homopolymer errors, these were identified by eye and if a possible AA always occurred with its parental sequences the read depth from the AA was added to that of the parental sequence. As a second step all amplicons with insufficient coverage was removed (the threshold was set to 110 for gDNA (and to 140 for cDNA, values in brackets) for primer combination 1, 70 (100) for primer combination 2, 100 (180) for primer combination 3 and 100 (140) for primer combination 4, see Additional file 1: Methods S2 for more details). Next, all sequences that had too few reads within an amplicon were deleted, this read depth was measured as percentage of the total read depth (varying from 1.1% to 3.0% depending on species and primer combination, see Additional file 1: Methods S2 for more details). All sequences that varied by 1–2 bp were identified and the read depth of the possible AA was only added to the parental sequences if the AA occurred only once in the entire data set, together with the parental sequence and when the read depth of the possible AA was less than half of the parental sequence. Chimeras and non-functional sequences were identified by eye and deleted from the data set. Possible chimeras were deleted from the data set only when they occurred with both parental sequences and when the read depth of the putative chimera was less than half of both parental sequences. Sequences that only occurred in one amplicon in the entire data set or in a single amplicon within an individual were deleted. Lastly low frequency sequences that were only amplified in cDNA were deleted since all cDNA sequences should also be found in the corresponding gDNA sample.

All sequences that remained after the strict filtering were considered TA. BLAST was used to determine which alleles had previously been published. When an allele was 100% identical to a previously published sequence and of the same length the allele was named according to the published sequence. If an allele was 100% identical to a previously published sequence but of different length the allele was given the name of the published sequence followed by ‘a’. Alleles that had not previously been published were given species specific names according to the recommended guide lines for naming MHC alleles [44]. All new sequences were deposited in GenBank (GenBank Acc nr KY303944-KY304003). TA in the cDNA samples, i.e. transcribed alleles, are hereafter called expressed alleles. Thirty-six samples were run in duplicates and the repeatability between duplicates was calculated as the percentage of total number of alleles amplified (i.e. the concatenated number of alleles using both duplicates as ‘total number’).

### Number and expression of MHC-I alleles

The use of multiple primer combinations enabled a detailed characterization of the total number of classical and non-classical MHC-I alleles per individual since the possibility of amplifying all alleles in an individual increases when different primer combinations are used [8, 42]. The number of alleles per individual was determined by combining the result from all four primer combinations. The amplification range of the four different primer combinations was calculated as the proportion of the total number of alleles amplified with all primer combinations. This was done separately for each individual and an average was calculated for classical and non-classical alleles in each species (Additional file 1: Table S2). We used primer combination 1 in the expression analysis since this primer combination amplified the majority of all the classical and non-classical alleles simultaneously. With the concatenated result from all four primer combinations we identified which alleles were expressed in each individual and in the expression analysis we only included individuals where primer combination 1 amplified the majority of all expressed alleles. Primer combination 1 fulfilled these strict criteria in six individuals (house sparrow, *n* = 3, tree sparrow, *n* = 3). The relative expression of each allele was estimated as the proportion of the total number of reads per individual. These six individuals expressed up to four classical and up to four non-classical alleles per individual, the maximum total number of expressed alleles was eight. In order to get an estimate of how many alleles that were highly expressed we set a custom threshold to define and separate highly expressed alleles from the remaining alleles based on the following reasoning: Given that there was a maximum of eight alleles per individual, each allele would with an even distribution contribute with 12.5% of the reads. We set the threshold for ‘high allelic expression’ to twice as high; hence highly expressed alleles should contribute with more than 25% of the reads in an individual.

### Statistical analysis and phylogenetic relationship

Statistical analyses were performed in SPSS (IBM SPSS Statistics 22). The differences between species regarding number of alleles and number of expressed alleles were determined with one-way ANOVAs. The differences in variation in expression between classical and non-classical genes were determined in two ways. First, when only expressed alleles were included in the model, Levine’s test of equal variance was used. Second, when also non-expressed alleles (i.e. alleles with zero read depth in cDNA sample) were included in the model the data was no longer normally distributed and hence the Brown-Forsythe variance test was used. In order to determine the phylogenetic relationship between MHC-I alleles both a maximum likelihood tree and a neighbor-net network were construed. The maximum likelihood tree was constructed with the RAxML software (version 7.0.4) using the GTRGAMMA model and 1000 bootstraps and illustrated with iTOL (version 3.4.3) [45]. The network was constructed with SplitsTree v. 4.14.4 [46] using a GTR model with the α parameter for gamma distribution set to 0.3450, which was recommended by jModelTest 2.1.10 [47] and 1000 bootstraps. Figures were produced in R, (version 2.15.3), using the built in packages barplot and plot [48].

## Results

### Phylogenetic relationship of putatively classical and non-classical MHC-I genes

MHC-I alleles were genotyped in 13 individuals, five house sparrows, three Spanish sparrows and five tree sparrows, using high-throughput sequencing of both gDNA (genomic DNA) and cDNA (complementary DNA, i.e. reverse transcribed RNA, as a measure of gene expression). The average read depth of true alleles (alleles that remained after filtering the HTS data) per individual varied between 413 and 990 reads for gDNA and between 528 and 1108 reads for cDNA (combining three or four primer combinations, for further details on read depth see Additional file 1: Table S3). The repeatability in genotyping between duplicates varied between 91% and 100% across primers (Additional file 1: Table S4). Putatively classical and non-classical MHC-I genes were found in all three Passer species, and the putatively non-classical alleles were identified by a 6 bp deletion in exon 3, as described previously for house sparrows. In total, in all three species, 129 alleles were identified (Additional file 1: Figure S2).

**Fig. 1:**
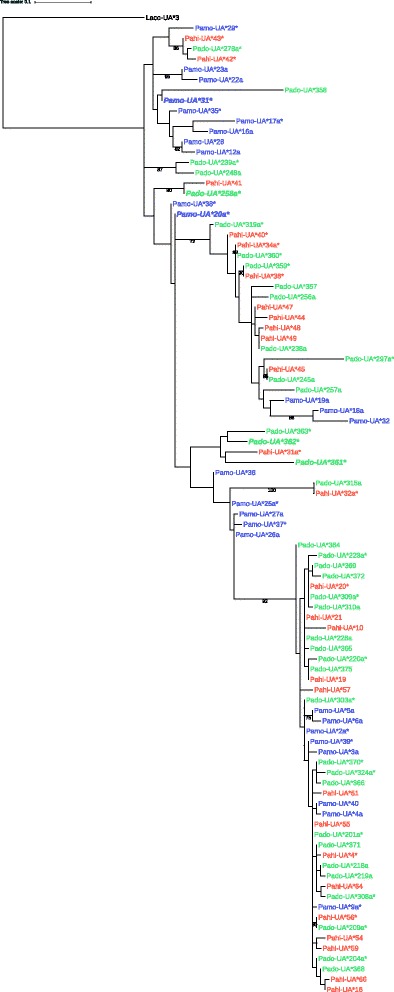
Maximum likelihood tree based on 94 MHC class I exon 3 nucleotide sequences from house sparrows (Pado; indicated in green), Spanish sparrows (Pahi; indicated in orange) and tree sparrows (Pamo; indicated in purple) amplified with primer combination 1. One MHC class I sequence (Acc nr KU169762) from *Lanius collaris* was used as outgroup. The tree was constructed with the RAxML software (version 7.0.4) using the GTRGAMMA model and 1000 bootstraps, displaying bootstrap values larger than 70%. *Stars* (*) indicates alleles that were found in both gDNA and cDNA (i.e. expressed alleles). The classical alleles that were identified as highly expressed are marked in bold and italic. All putatively non-classical alleles are found in the lower cluster, with no clustering based on species, whereas the putatively classical alleles do not form a distinct cluster

The phylogenetic relationship of the putatively classical and non-classical MHC-I genes placed all putatively non-classical alleles in a distinct cluster, in the maximum likelihood tree and the neighbor-net network with high bootstrap support, 92 and 93 respectively (Fig. 1, Additional file 1: Figure S3). This shows that the separation of putatively classical and non-classical MHC-I genes predates the speciation of the investigated sparrow species. The putatively non-classical alleles have short branches and are hence highly similar, whereas the putatively classical alleles are much more variable, and this is further supported by the higher nucleotide diversity and amino acid sequence per nucleotide sequence for classical alleles (Additional file 1: Table S5). Moreover, no clear phylogenetic separation based on expression could be seen since expressed alleles were found across the phylogenetic tree (Fig. 1, Additional file 1: Figure S3).

### Number of putatively classical and non-classical MHC-I alleles among sparrow species

**Fig. 2:**
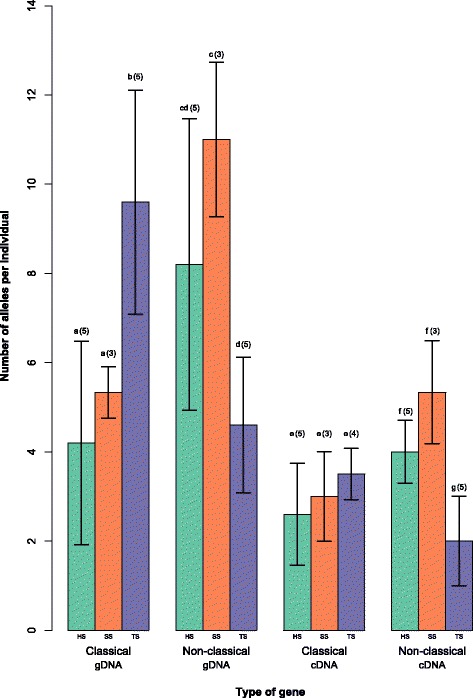
Average numbers of putatively classical and non-classical MHC-I alleles per individual in gDNA (genomic DNA) and cDNA (i.e. expressed alleles) in house sparrows (HS; indicated in *green*), Spanish sparrows (SS; indicated in orange) and tree sparrows (TS; indicated in *purple*), for further details see Additional file 1: Table S4. Tukey posthoc test was used to determine differences between the groups, significance is indicated by letters (a-g; *p* < 0.05) and number of individuals is reported within brackets

The number of putatively classical gDNA alleles per individual varied significantly between species (*F* = 8.418, *p* = 0.007), as did the number of putatively non-classical gDNA alleles (*F* = 7.003, *p* = 0.013, Fig. 2, Additional file 1: Table S6, Table S7). The highest number of gDNA alleles in a house sparrow was seven putatively classical and 13 putatively non-classical alleles, in a Spanish sparrow six and 12 and in a tree sparrow 14 and seven. The number of expressed putatively non-classical alleles varied significantly between the three species (*F* = 13.018, *p* = 0.002, Fig. 2, Additional file 1: Table S6, Table S7), whereas no such difference was seen for the number of expressed putatively classical alleles. The highest number of expressed alleles in house sparrows was four putatively classical and five putatively non-classical, in Spanish sparrows four and six and in tree sparrows four and three.

### Variance in expression of classical and non-classical MHC-I genes in house sparrows and tree sparrows

**Fig. 3:**
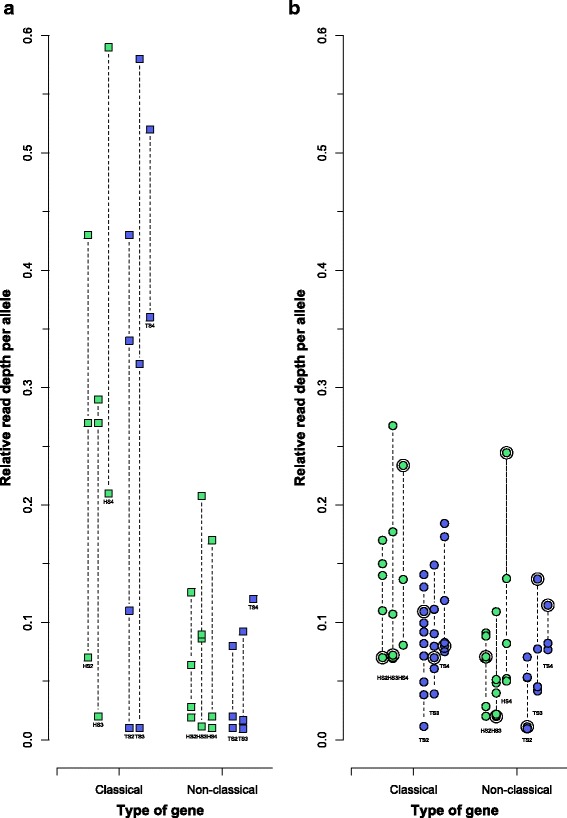
Variance in relative read depth per allele of putatively classical and non-classical alleles in three house sparrow (HS2, HS3, and HS4; indicated in *green*) and three tree sparrow individuals (TS2, TS3 and TS4; indicated in *purple*). **a** In cDNA (*squares*) there is a significant difference in variance in expression (measured as relative read depth per allele) between putatively classical and non-classical alleles (Levine’s test: *F* = 5.20, *p* = 0.005). **b** In gDNA (*circles*) there is no difference in relative read depth per allele. The highest expressed allele in cDNA does not correspond to the allele with highest relative read depth in gDNA. These highly expressed alleles are marked with a *circle* in the gDNA plot

Putatively classical MHC-I genes had a significantly higher variance in expression, measured as relative read depth, than putatively non-classical genes (Levine’s test: *F* = 5.20, *p* = 0.005; Fig. 3a). This difference between putatively classical and non-classical genes is still present when non-expressed alleles are included in the model (Brown-Forsythe variance test: F = 6.62, *p* = 0.012). The large variance in expression among putatively classical genes suggests that only a subset of these genes is highly expressed. The variance in relative read depth was not significantly different between putatively classical and non-classical genes in gDNA (Levine’s test: *F* = 0.883, *p* = 0.455, Fig. 3b), and hence there is no bias in amplification efficiency of the primers between the genes. The difference in variance seen for expressed putatively classical and non-classical genes can therefore not be a result of biased amplification of certain alleles. Two tree sparrow individuals were run in duplicates and the relative read depth is highly similar between duplicates, (Additional file 1: Table S8). Only three house sparrows and three tree sparrows fulfilled the criteria to be included in the expression analyses, i.e. that the majority of all the identified expressed alleles were amplified with primer combination 1 (for further details see Additional file 1: Table S9, Figure S4). In these six individuals at most two putatively classical alleles were highly expressed per individual suggesting that only a single putatively classical gene is highly expressed in sparrows (assuming heterozygosity). None of the putatively non-classical alleles were highly expressed.

## Discussion

The subdivision of MHC-I genes into classical, highly polymorphic genes that present peptides to T-cells, and non-classical genes, less polymorphic genes that do not present peptides to T-cells, have been reported in many mammal species and also in several bird species [3–5, 10, 11, 19–21, 27–30]. However, classical and non-classical genes have not yet been confirmed in songbirds in the largest bird order Passeriformes, though such subdivision is likely since non-classical genes have been identified in other bird species from several different orders [25–30]. In the present study we found high numbers of MHC-I gene copies in sparrows; the maximum number of MHC-I alleles per individual that we identified was 21 (i.e sparrows have eleven or more MHC-I gene copies), though only about 50% of these alleles were expressed. In the maximum likelihood tree we identified one distinct strongly supported cluster (bootstrap = 92 and for the neighbor-net network the corresponding the bootstrap = 93) containing alleles (from all three species) with low polymorphism and a 6 bp deletion (putatively non-classical genes). The remaining alleles were more polymorphic and found in non-significantly supported groups (putatively classical genes). Several previous studies have reported considerably lower diversity estimates and lower rates of non-synonymous substitutions in the peptide binding region of putatively non-classical alleles compared with putatively classical alleles in house sparrows [35, 36, 49], and we found similar results in our data from three different sparrow species considering nucleotide diversity. Moreover, the analyses of gene expression showed that the polymorphic group with the putatively classical genes had variable expression, that is, some alleles were highly expressed while others had low expression. Strikingly, at most two alleles among these putatively classical MHC-I alleles were ever highly expressed in each individual. In contrast, the group with putatively non-classical genes had more uniformly low expression. Taken together, these results strongly indicate that we have identified classical and non-classical MHC-I genes in sparrows.

### Phylogenetic ages of classical and non-classical genes

The phylogenetic reconstruction of sparrow MHC-I alleles places the non-classical genes in a single well-supported cluster, confirming that the subdivision of these putatively classical and non-classical genes predates the separation of the investigated sparrow species. This shows that orthologous gene copies of classical and non-classical genes have persisted in sparrows over several speciation events in a time frame of at least 7 million years. Classical (MHC-B) and non-classical (MHC-Y) genes have also been reported among a wide range of birds in the order Galliformes, including chicken, turkey and golden pheasant, species that split 28–40 million years ago, and non-classical alleles in chicken and turkey form a gene specific cluster [4, 20, 21, 50–52]. It is possible that these different sets of paralogous genes are even older and evolved in an ancient common ancestor of galliforms more than 65 million years ago [53]. However, non-classical genes in sparrows of the order Passeriformes and species within the order Galliformes are not likely to be orthologous, presently available data suggest that the non-classical genes originate from more recent duplications of classical genes, a pattern seen also among distantly related mammals [14, 30]. Though, orthologous clusters of classical and non-classical MHC-I genes among relatively closely related mammals has been seen in hominids and here the classical and non-classical genes even cluster by locus [10]. Classical (HLA-A, -B and -C) and non-classical (HLA-E, -F and -G) MHC-I genes in human and chimpanzee (*Pan troglodytes*), species that diverged 6 to 7 million years ago, form gene specific clusters at each of these six loci (A-G).

### Number of MHC alleles in gDNA and cDNA in three sparrow species

We found no difference in the copy number of classical and non-classical alleles in the genome between house sparrows and Spanish sparrows (species that split 3 million years ago) but there was a difference in allele copy number relative to the tree sparrows, which diverged earlier (7 million years ago). This difference in gene copy number could have originated in two different ways, first the divergence time of house sparrows and Spanish sparrows may be too short for gene copy number to evolve while tree sparrows that are more distantly related have evolved further. Tree sparrows have a higher number of classical gDNA alleles and a lower number of non-classical gDNA alleles than house sparrows and Spanish sparrows. Alternatively, house sparrow and Spanish sparrow could have experienced different selection and lost classical gene copies. Without knowing the proportion of classical and non-classical genes in ancestral sparrows it is impossible to determine how this gene copy variation occurred. Moreover, there are certain problems associated with co-amplifying multiple genes at the same time which makes it more difficult to determine the exact number of genes and possible copy number variation [54, 55]. Here we have tried to overcome this problem by using several primer combinations.

The number of expressed alleles (cDNA) varied less between species than the number of alleles in the genome and, interestingly, there was no significant difference in any species comparisons in number of expressed classical alleles. One explanation could be that the number of expressed genes are more conserved than the total number of genes in the genome and there may be selection for expressing a certain number of genes, e.g. expressing an optimal number of classical genes [56]. There was a significant difference in the number of expressed non-classical alleles among species; tree sparrows expressed fewer non-classical alleles than both house sparrows and Spanish sparrows. It is interesting to note that tree sparrows, which express significantly lower numbers of non-classical alleles, also have a lower number of non-classical alleles in the genome.

### Variation in expression of classical and non-classical MHC-I alleles

The variance in gene expression was larger in putatively classical than non-classical MHC-I genes in sparrows. This finding is consistent with the existence of ‘major’ (highly expressed) and ‘minor’ (low expressed) loci for classical but not for non-classical MHC-I genes in sparrows, as previously reported for species within the order Galliformes [22, 23]. The highest numbers of classical alleles per individual in sparrows were 14 in gDNA but out of these only two alleles were highly expressed. Two highly expressed classical MHC-I alleles have been reported for several bird species in the order Galliformes (e.g. chicken and Japanese quail (*Coturnix japonica*)) and also in mallards belonging to the order Anseriformes. In chicken, Japanese quail and mallard the MHC genomic regions have been characterized and each species has a single major classical MHC-I locus, that is, one classical gene that is highly expressed [27, 57–59]. In all these species, the highly expressed gene is located next to the TAP gene (Transporter associated with antigen processing) and co-evolutionary processes between TAP and MHC is thought to explain why only a single MHC-I gene is highly expressed [4, 31, 57, 60–62]. Our findings on classical MHC-I genes in house sparrows and tree sparrows agree well with these previous findings from other birds, though with our data we cannot determine with certainty if sparrows have one single major classical MHC-I gene. It is possible that the six sparrows are homozygous for two classical MHC-I genes that are highly expressed, even though this is unlikely since heterozygosity is much more common than homozygosity at classical MHC-I loci. Alternatively, different genes could be highly expressed in the six individuals or the two alleles of one gene could be differently expressed, meaning that two genes could be highly expressed. It would be interesting to study this further and to determine if the highly expressed alleles belong to the same gene and if this gene is located next to TAP. Since we set strict criteria for including individuals in the expression analysis we could only investigate six of the individuals. Future analysis of more individuals would help determining how general our results are.

In our study of expression of MHC-I genes we only estimated gene expression in blood. Blood to some extent represent gene expression in different tissues but we do not claim that our results should be extrapolated to be representative for expression in all tissues. Non-expressed genes in our study could for example have specific expression under other conditions, in other tissues or in birds of different age classes. Chen et al. [30] recently characterized the genomic MHC region in the crested ibis (*Nipponia nippon*) and reported considerable differences in gene expression of five different MHC-I genes between tissues. Interestingly one particular MHC-I gene was highly expressed in all tissues in the crested ibis and this gene was the only gene situated in the core MHC genomic region [30]. Expression in blood was however not reported in the crested ibis.

## Conclusions

We have studied the highly duplicated MHC-I gene family in three species of sparrows and based on phylogeny and gene expression patterns found strong indications for the existence of classical and non-classical MHC-I genes. This subdivision of genes has previously been reported in many groups of vertebrates, for example in galliform birds and hominids, but never in songbirds. A majority of the sparrow MHC-I genes are putatively non-classical; hence they are presumably not involved in T-cell mediated immunity. Such a distinctly separated phylogenetic cluster of putatively non-classical genes is rarely found among songbirds, and within songbirds non-classical genes could be a unique feature for sparrows. However, we find it more likely that there are groups of non-classical MHC-I genes in most songbirds but that they often are missed. Therefore, it would be valuable if future studies of MHC-I in songbirds investigated the existence of putatively non-classical MHC-I genes, preferably using gene expression. Future ecological and evolutionary studies of MHC-I in wild birds would gain from considering the existence of classical and non-classical genes, since these two types of MHC-I genes have different functions.

## Additional file

Additional file 1: Method S1. Extended methods for creating the Passer maximum clade credibility tree. **Method S2.** Extended filtering protocol for treating the high-throughput amplicon data. **Table S1.** Detailed information regarding the primers used for high-throughput amplicon sequencing. **Table S2.** Comparison of how efficient the four different primer combinations used in this study amplified MHC-I alleles. **Table S3.** Read depth per individual before and after filtering of the high-throughput amplicon data. **Table S4.** Comparison of the reputability between duplicated samples sequenced with 454 amplicon sequencing. **Table S5.** Diversity measurements of putatively classical and non-classical alleles. Calculated for all gDNA alleles, expressed and non-expressed alleles separately. **Table S6.** The number of putatively classical and non-classical MHC-I alleles identified, per individual, in gDNA and cDNA, for the 13 sparrow individuals used in this study. **Table S7.** List of the different alleles, both classical and non-classical, amplified in each individual. **Table S8.** Comparison of the relative read depth per allele between two duplicated tree sparrow individuals that were used for the expression analysis. **Table S9.** Number of expressed MHC-I alleles identified in the three house sparrow and three tree sparrow individuals used for the expression analysis. **Figure S1.** Schematic overview of MHC-I exon 3 displaying the different locations for all primers used in this study. **Figure S2.** Alignmnet of the 129 MHC-I concatenated exon 3 alleles identified. **Figure S3.** Neighbor-net network displaying the 94 MHC-I alleles amplified with primer combination 1. **Figure S4.** Comparison of the proportion of reads per allele between gDNA and cDNA in the three house sparrow and three tree sparrow individuals selected for the expression analysis. (DOCX 5357 kb)

## References

[1] Murphy K, Travers P, Walport M (2008). Janeway’s immunobiology.

[2] Neefjes J, Jongsma ML, Paul P, Bakke O (2011). Towards a systems understanding of MHC class I and MHC class II antigen presentation. Nat Rev Immunol.

[3] Shiina T, Hosomichi K, Inoko H, Kulski JK (2009). The HLA genomic loci map: expression, interaction, diversity and disease. J Hum Genet.

[4] Kaufman J, Milne S, Göbel TW, Walker BA, Jacob JP, Auffray C (1999). The chicken B locus is a minimal essential major histocompatibility complex. Nature.

[5] Lunney JK, Ho CS, Wysocki M, Smith DM (2009). Molecular genetics of the swine major histocompatibility complex, the SLA complex. Dev Comp Immunol.

[6] Westerdahl H (2007). Passerine MHC: genetic variation and disease resistance in the wild. J Ornithol.

[7] Sepil I, Moghadam HK, Huchard E, Sheldon BC, Kuduk K, Babik W (2012). Characterization and 454 pyrosequencing of major histocompatibility complex class I genes in the great tit reveal complexity in a passerine system. BMC Evol Biol.

[8] O’Connor EA, Strandh M, Hasselquist D, Nilsson J, Westerdahl H (2016). The evolution of highly variable immunity genes across a passerine bird radiation. Mol Ecol.

[9] Biedrzycka A, O’Connor E, Sebastian A, Migalska M, Radwan J, Zając T, et al. Extreme MHC class I diversity in the sedge warbler (*Acrocephalus schoenobaenus*); selection patterns and allelic distributions suggest that different genes have different functions. BMC Evol Biol. 2017. In press. 10.1186/s12862-017-0997-9 PMC5497381 28679358

[10] Adams E, Parham P (2001). Species-specific evolution of MHC class I genes in the higher primates. Immunol Rev.

[11] Velten F, Rogel-Gaillard C, Renard C, Pontarotti P, Tazi-Ahnini R, Vaiman M (1998). A first map of the porcine major histocompatibility complex class I region. Tissue Antigens.

[12] Shawar S, Vyas J (1994). Antigen presentation by major histocompatibility complex class IB molecules. Annu Rev Immunol.

[13] Rodgers JR, Cook RG (2005). MHC class Ib molecules bridge innate and acquired immunity. Nat Rev Immunol.

[14] Hughes ALL, Nei M (1989). Evolution of the major histocompatibility complex: independent origin of nonclassical class I genes in different groups of mammals. Mol Biol Evol.

[15] Ishitani A, Sageshima N, Lee N, Dorofeeva N, Hatake K, Marquardt H (2003). Protein expression and peptide binding suggest unique and interacting functional roles for HLA-E, F, and G in maternal-placental immune recognition. J Immunol.

[16] Diefenbach A, Raulet DH (2002). The innate immune response to tumors and its role in the induction of T-cell immunity. Immunol Rev.

[17] Robinson J, Halliwell J, Hayhurst JD, Flicek P, Parham P, Marsh SGE (2015). The IPD and IMGT/HLA database: Allele variant databases. Nucleic Acids Res.

[18] Apps R, Meng Z, Del Prete GQ, Lifson JD, Zhou M, Carrington M (2015). Relative Expression Levels of the HLA Class-I Proteins in Normal and HIV-Infected Cells. J Immunol.

[19] Briles WE, Goto RM, Auffray C, Miller MM (1993). A polymorphic system related to but genetically independent of the chicken major histocompatibility complex. Immunogenetics.

[20] Chaves LD, Krueth SB, Reed KM (2007). Characterization of the turkey MHC chromosome through genetic and physical mapping. Cytogenet Genome Res.

[21] Zeng Q, Zhong G, He K, Sun D, Wan Q (2016). Molecular characterization of classical and nonclassical MHC class I genes from the golden pheasant (*Chrysolophus pictus* ). Immunogenetics.

[22] Kaufman J, Jacob J, Shaw I, Walker B, Milne S, Beck S (1999). Gene organisation determines evolution of function in the chicken MHC. Immunol Rev.

[23] Kaufman J (1999). Co-evolving genes in MHC haplotypes: the “rule” for nonmammalian vertebrates?. Immunogenetics.

[24] Wallny H-J, Avila D, Hunt LG, Powell TJ, Riegert P, Salomonsen J (2006). Peptide motifs of the single dominantly expressed class I molecule explain the striking MHC-determined response to Rous sarcoma virus in chickens. Proc Natl Acad Sci U S A.

[25] Afanassieff M, Goto RM, Ha J, Sherman MA, Zhong L, Auffray C (2001). At least one class I gene in restriction fragment pattern-Y (Rfp-Y), the second MHC gene cluster in the chicken, is transcribed, polymorphic, and shows divergent specialization in antigen binding region. J Immunol.

[26] Hunt HD, Goto RM, Foster DN, Bacon LD, Miller MM (2006). At least one YMHCI molecule in the chicken is alloimmunogenic and dynamically expressed on spleen cells during development. Immunogenetics.

[27] Moon DA, Veniamin SM, Parks-Dely JA, Magor KE (2005). The MHC of the duck (*Anas platyrhynchos*) contains five differentially expressed class I genes. J Immunol.

[28] Cloutier A, Mills JA, Baker AJ (2011). Characterization and locus-specific typing of MHC class I genes in the red-billed gull (*Larus scopulinus*) provides evidence for major, minor, and nonclassical loci. Immunogenetics.

[29] Buehler DMDM, Verkuil YIYI, Tavares ESES, Baker AJAJ (2013). Characterization of MHC class i in a long-distance migrant shorebird suggests multiple transcribed genes and intergenic recombination. Immunogenetics.

[30] Chen L-C, Lan H, Sun L, Deng Y-L, Tang K-Y, Wan Q-H (2015). Genomic organization of the crested ibis MHC provides new insight into ancestral avian MHC structure. Sci Rep.

[31] Mesa CM, Thulien KJ (2004). Moon D a, Veniamin SM, Magor KE. The dominant MHC class I gene is adjacent to the polymorphic TAP2 gene in the duck, *Anas platyrhynchos*. Immunogenetics.

[32] Ohno S (1970). Evolution by Gene Duplication.

[33] Eirin-Lopez JM, Rebordions L, Rooney AP, Rozas J (2012). The Birth-and-Death Evolution of Multigene Families Revisited. Genome Dyn..

[34] Nei M, Gu X, Sitnikova T (1997). Evolution by the birth-and-death process in multigene families of the vertebrate immune system. Proc Natl Acad Sci National Acad Sciences.

[35] Karlsson M, Westerdahl H (2013). Characteristics of MHC Class I Genes in House Sparrows *Passer domesticus* as Revealed by Long cDNA Transcripts and Amplicon Sequencing. J Mol Evol.

[36] Bonneaud C, Sorci G, Morin V, Westerdahl H, Zoorob R, Wittzell H (2004). Diversity of Mhc class I and IIB genes in house sparrows (*Passer domesticus*). Immunogenetics.

[37] Mullarney K, Svensson L, Zetterström D, Grant PJ (2006). Bird guide. The most complete field guide to the birds of britain and europe.

[38] Jetz W, Thomas GH, Joy JB, Hartmann K, Mooers AO (2012). The global diversity of birds in space and time. Nature.

[39] A Global Phylogeny of Birds. http://birdtree.org. Accessed 25 Nov 2015.

[40] Sambrook J, Fritsch EFMT (1989). Molecular cloning: a laboratory manual.

[41] Chiari Y, Galtier N (2011). RNA extraction from sauropsids blood: evaluation and improvement of methods. Amphibia-Reptilia.

[42] Westerdahl H, Wittzell H, von Schantz T, Bensch S (2004). MHC class I typing in a songbird with numerous loci and high polymorphism using motif-specific PCR and DGGE. Heredity (Edinb).

[43] Kloch A, Babik W, Bajer A, Siński E, Radwan J (2010). Effects of an MHC-DRB genotype and allele number on the load of gut parasites in the bank vole Myodes glareolus. Mol Ecol.

[44] Klein J, Bontrop RE, Dawkins RL, Erlich HA, Gyllensten UB, Heise ER (1990). Nomenclature for the major histocompatibility complexes of different species: a proposal. Immunogenetics.

[45] Letunic I, Bork P (2016). Interactive tree of life (iTOL) v3: an online tool for the display and annotation of phylogenetic and other trees. Nucleic Acids Res..

[46] Huson DH, Bryant D (2006). Application of phylogenetic networks in evolutionary studies. Mol Biol Evol.

[47] Posada D (2008). jModelTest: Phylogenetic model averaging. Mol Biol Evol.

[48] RDC T, R Core Team (2014). R: A Language and Environment for Statistical Computing.

[49] Borg AA, Pedersen SA, Jensen H, Westerdahl H (2011). Variation in MHC genotypes in two populations of house sparrow (*Passer domesticus*) with different population histories. Ecol Evol.

[50] Reed KM, Bauer MM, Monson MS, Benoit B, Chaves LD, O’Hare TH (2011). Defining the turkey MHC: identification of expressed class I- and class IIB-like genes independent of the MHC-B. Immunogenetics.

[51] Dimcheff DE, Drovetski SV, Mindell DP (2002). Phylogeny of Tetraoninae and other galliform birds using mitochondrial 12S and ND2 genes. Mol Phylogenet Evol.

[52] Van Tuinen M, Dyke GJ (2004). Calibration of galliform molecular clocks using multiple fossils and genetic partitions. Mol Phylogenet Evol.

[53] Jarvis E, Mirarab S, Aberer A, Li B, Houde P, Li C (2014). Whole-genome analyses resolve early branches in the tree of life of modern birds. Science..

[54] Burri R, Promerová M, Goebel J, Fumagalli L (2014). PCR-based isolation of multigene families: lessons from the avian MHC class IIB. Mol Ecol Resour.

[55] Gaigher A, Burri R (2016). Family-assisted inference of the genetic architecture of major histocompatibility complex variation.

[56] Milinski M (2006). The Major Histocompatibility Complex, Sexual Selection, and Mate Choice. Annu Rev Ecol Evol Syst.

[57] Kaufman J, Völk H, Wallny HJ (1995). A “minimal essential Mhc” and an “unrecognized Mhc”: two extremes in selection for polymorphism. Immunol Rev.

[58] Shiina T, Hosomichi K, Hanzawa K (2006). Comparative genomics of the poultry major histocompatibility complex. Anim Sci J.

[59] Fleming-canepa X, Jensen SM, Christine M, Diaz-satizabal L, Roth AJ, Parks-dely JA (2016). Extensive Allelic Diversity of MHC Class I in Wild Mallard Ducks. J Immunol.

[60] Shiina T, Oka A, Imanishi T, Hanzawa K, Gojobori T, Watanabe S (1999). Multiple class I loci expressed by the quail Mhc. Immunogenetics.

[61] Shiina T, Shimizu S, Hosomichi K, Kohara S, Watanabe S, Hanzawa K (2004). Comparative genomic analysis of two avian (quail and chicken) MHC regions. J Immunol.

[62] Walker BA, Hunt LG, Sowa AK, Skjødt K, Göbel TW, Lehner PJ (2011). The dominantly expressed class I molecule of the chicken MHC is explained by coevolution with the polymorphic peptide transporter (TAP) genes. Proc Natl Acad Sci U S A.

[63] Drews A, Strandh M, Råberg L, Westerdahl H. Data from: Expression and phylogenetic analyses reveal paralogous lineages of putatively classical and non-classical MHC-I genes in three sparrow species (*Passer*). Dryad Digital Repository. http://dx.doi.org/10.5061/dryad.79t4b. 10.1186/s12862-017-0970-7 PMC5485651 28651571

