# Supplementary material for: Expression and phylogenetic analyses reveal paralogous lineages of putatively classical and non-classical MHC-I genes in three sparrow species (Passer)
Source: BMC Evol Biol. 2017 Jun 26;17:152. doi: 10.1186/s12862-017-0970-7 (PMC5485651; doi:10.1186/s12862-017-0970-7)
Supplement: Additional file 1: Method S1. — Extended methods for creating the Passer maximum clade credibility tree. Method S2. Extended filtering protocol for treating the high-throughput amplicon data. Table S1. Detailed information regarding the primers used for high-throughput amplicon sequencing. Table S2. Comparison of how efficient the four different primer combinations used in this study amplified MHC-I alleles. Table S3. Read depth per individual before and after filtering of the high-throughput amplicon data. Table S4. Comparison of the reputability between duplicated samples sequenced with 454 amplicon sequencing. Table S5. Diversity measurements of putatively classical and non-classical alleles. Calculated for all gDNA alleles, expressed and non-expressed alleles separately. Table S6. The number of putatively classical and non-classical MHC-I alleles identified, per individual, in gDNA and cDNA, for the 13 sparrow individuals used in this study. Table S7. List of the different alleles, both classical and non-classical, amplified in each individual. Table S8. Comparison of the relative read depth per allele between two duplicated tree sparrow individuals that were used for the expression analysis. Table S9. Number of expressed MHC-I alleles identified in the three house sparrow and three tree sparrow individuals used for the expression analysis. Figure S1. Schematic overview of MHC-I exon 3 displaying the different locations for all primers used in this study. Figure S2. Alignmnet of the 129 MHC-I concatenated exon 3 alleles identified. Figure S3. Neighbor-net network displaying the 94 MHC-I alleles amplified with primer combination 1. Figure S4. Comparison of the proportion of reads per allele between gDNA and cDNA in the three house sparrow and three tree sparrow individuals selected for the expression analysis. (DOCX 5357 kb) [file 12862_2017_970_MOESM1_ESM.docx]

**Method S1.** *Maximum clade credibility tree*

In order to determine when house sparrow, Spanish sparrow and tree sparrow separated a phylogenetic tree for 23 Passer species (*Passer ammodendri, Passer domesticus, Passer hispaniolensis, Passer pyrrhonotus, Passer castanopterus, Passer rutilans, Passer flaveolus, Passer moabiticus, Passer iagoensis, Passer rufocinctus, Passer insularis, Passer motitensis, Passer melanurus, Passer griseus, Passer swainsonii, Passer gongonensis, Passer suahelicus, Passer diffusus, Passer simplex, Passer montanus, Passer luteus, Passer euchlorus, Passer eminibey*) was constructed with data from the Bird Tree website (http://birdtree.org/) [1]. Jetz *et al*. [1] generated a phylogenetic tree for nearly all bird species using Bayesian phylogenetic methods. One thousand trees from the posterior distribution were downloaded for the 23 Passer species of interest, in addition to *Cyanistes caeruleus*as an outgroup, using the subset tool on the Bird Tree website. These trees were based on a composite of existing genetic data built upon a backbone tree from Hackett *et al*. [2] combined with topological and fossil constraints. The 1000 trees were then used to create a maximum clade credibility tree in the program TreeAnnotator within Beast v1.7 [3].

**Method S2.** *Filtering* *steps*

The program jMHC was used to extract the raw 454-data [4]. Only sequences with complete primers and tags, both forward and reverse, were extracted. In order to control that all sequences that jMHC returned were unique the web-applications seqeqseq (http://mbio-serv2.mbioekol.lu.se/apps/seqeqseq.html) was used, followed by mergeMatrix (http://mbio-serv2.mbioekol.lu.se/apps/mergeMatrix.html). Since most AA will occur at low frequencies sequences with only one read were deleted. Homopolymer errors are common in 454-sequencing [5]. In order to reduce these errors the sequences were aligned in BioEdit [6], based on conserved amino acid positions, and insertions or deletions in connection to homopolymers were identified. If the suspected homopolymer error occurred with its parent sequence the AA was removed and its read number was added to the parent sequences. First, amplicons with insufficient coverage were deleted and this deletion threshold was set by plotting the total read depth for all amplicons within a species, primer combination and sample type (gDNA/cDNA). The thresholds was set to (set to (cDNA values in brackets) 110 (140) for primer combination 1, 70 (100) for primer combination 2, 100 (180) for primer combination 3 and 100 (140) for primer combination 4. The remaining steps of the filtering were done as suggested by Galan *et al*. [7], separately for each primer combination, bird species and sample type. A global threshold for the per amplicon frequency of specific sequences was determined using the web-application popMatrix (http://mbio-serv2.mbioekol.lu.se/apps/popMatrix.html). The sample duplicates were used to determine the best global threshold to separate TA and AA. The global threshold for keeping sequences in house sparrows was set at (cDNA in brackets) 1.2% (1.1%) for primer combination 1, 1.2% (2.1%) for primer combination 2, 2.3% (2.0%) for primer combination 3 and 1.8% (2.3%) for primer combination 4. In Spanish sparrows the global threshold for keeping sequences was 2.1% (1.7%) for primer combination 1, 1.8% for primer combination 2, 2.2% (2.4%) for primer combination 3 and 2.3% (2.5%) for primer combination 4. In tree sparrows the global threshold for keeping sequences was 2.6% (1.9%) for primer combination 1, (1.2%) for primer combination 2, 3.0% (2.6%) for primer combination 3 and 3.0% (2.7%) for primer combination 4. Sequences that were found in both gDNA and cDNA were considered verified, meaning that cDNA sequences in particular were kept in the data set even if their frequencies were below the threshold. The final step of filtering was to align all sequences based on conserved amino acid positions. One alignment per individual, primer combination and sample type was produced. The alignment was inspected by eye for possible chimeras and nucleotide substitutions (*i.e.* a sequence that varied by one or two bases from another sequences). Chimeras were identified and deleted if they occurred with both its parental sequences and if the read number of the chimeric sequence was less than half of both parental sequences. The criteria for 1-2 nucleotide substitutions was identical, these sequences were deleted if they only occurred once in the entire data set, only together with a parental sequence and if the read number of this sequence was less than half of the parental sequence. Identified nucleotide substitution errors were removed and the read number was added to the parental sequence. Finally, non-functional sequences were deleted from the data set.

The pre-filtered data from all four primer combinations, sample types (gDNA/cDNA) and individuals was combined for the next filtering steps. First, all sequences that were only amplified in a single amplicon in the entire data set were deleted. Second, all sequences that were only amplified in a single amplicon within a single individual were deleted if the read depth was close to the threshold and the sequence was satisfactory amplified in a different individual. Third, all sequences that had a per amplicon frequency below the set threshold were checked. This was done within a single individual; if the sequence had been amplified satisfactory in another amplicon belonging to the same individual the sequences were kept otherwise it was deleted.

Finally, all sequences that were only amplified in cDNA were deleted, since these alleles should also occur in gDNA (9 such putative AA were detected in 7 individuals (2 house sparrows, 1 Spanish sparrow, 4 tree sparrows), having 4 (4.3%), 5 (2.2%), 7 (4.4%), 9 (3.8%), 9 (4.3%), 9 (5.1%), 11 (6.0%), 12 (6.4%) and 16 (5.4%) reads. 2 alleles were classical and 7 were non-classical).

T**able S1** Four different primer combinations were used for the 454 amplicon sequencing. The combinations included one of four forward primers (F) combined with one of two reverse primers (R). Long-rv3 and Short-rv3 were designed to amplify classical (Long) and non-classical (Short) alleles in house sparrow. HNalla were originally designed for MHC-I in great reed warbler but amplifies satisfactory also in other songbird species. FWD3 and RVS3b were designed to amplify MHC-I in a large number of songbird species across the Passeriformes tree.

| Primer combination | Primer name | Primer sequences | Amplicon size (bp) | Reference |
| --- | --- | --- | --- | --- |
| 1 | HNalla (F) | 5' TCCCCACAGGTCTCCACAC | 185-191 | [8] |
| 1 | RVS3b (R) | 5' TGGTTGCGAGTTTACGGYTRTG |  | [9] |
| 2 | FWD3 (F) | 5' GGCAGACGTGCTYCWRGTAATT | 220-226 | [9] |
| 3 | Long (F) | 5' GTCTCCACACTGTACAGYGGC | 229-232 | [10] |
| 4 | Short (F) | 5' GTCTMCACACGAGGTTGCGAG | 226 | [10] |
| 2,3,4 | Rv3 (R) | 5' TGCGCTCCAGCTCCYTCTGCC |  | [10] |

**Table S2** Comparison of how efficient the different primer combinations amplified all MHC-I alleles, from genomic DNA (gDNA) and RNA (cDNA). Calculated as the proportion of the total number of alleles per individual that each primer combination amplified. Reported is the average proportion ±SD per species.

|  | House sparrow | | | | Spanish sparrow | | | | Tree sparrow | | | |
| --- | --- | --- | --- | --- | --- | --- | --- | --- | --- | --- | --- | --- |
|  | gDNA | | cDNA | | gDNA | | cDNA | | gDNA | | cDNA | |
|  | Classical | Non-classical | Classical | Non-classical | Classical | Non-classical | Classical | Non-classical | Classical | Non-classical | Classical | Non-classical |
| Primer combination 1 | 1.00  ±0.00 | 0.88  ±0.13 | 0.95  ±0.11 | 0.77  ±0.34 | 1.00  ±0.00 | 0.52  ±0.09 | 1.00  ±0.00 | 0.50  ±0.00 | 0.79  ±0.09 | 0.66  ±0.34 | 0.77  ±0.16 | 1.00  ±0.00 |
| Primer combination 2 | - | 0.94  ±0.05 | - | 0.75  ±0.15 | - | 0.94  ±0.10 | - | 0.67  ±0.00 | 0.22  ±0.09 | 0.96  ±0.07 | 0.31  ±0.34 | 1.00  ±0.00 |
| Primer combination 3 | 0.78  ±0.20 | - | 0.68  ±0.34 | - | 0.82  ±0.17 | - | 0.81  ±0.17 | - | 0.71  ±0.11 | - | 0.58  ±0.10 | - |
| Primer combination 4 | - | 0.98  ±0.07 | - | 0.94  ±0.13 | - | 0.97  ±0.05 | - | 0.94  ±0.10 | 0.25  ±0.07 | 0.94  ±0.13 | 0.23  ±0.16 | 1.00  ±0.00 |

**Table S3** Read depth per individual, for the four different primer combinations used. Separated by species and by sample type; gDNA (a) and cDNA (b). Read depth is displayed before filtering (raw data) and after all filtering steps (filtered data). N.I indicates samples not included in the sequencing and - indicates samples were the sequencing failed.

| (a) | **Primer combination 1** | | **Primer combination 2** | | | **Primer combination 3** | | | | **Primer combination 4** | | |
| --- | --- | --- | --- | --- | --- | --- | --- | --- | --- | --- | --- | --- |
| Individual | Before filtering | After filtering | | Before filtering | After filtering | | Before filtering | After filtering | Before filtering | | After filtering |  |
| House sparrow 1 | 320 | 169 | | 233 | 118 | | 283 | 176 | 221 | | 125 |  |
| House sparrow 1_Rep | 228 | 126 | | N.I | N.I | | 272 | 161 | N.I | | N.I |  |
| House sparrow 2 | 312 | 187 | | - | - | | 210 | 150 | 192 | | 105 |  |
| House sparrow 2_Rep | 278 | 156 | | N.I | N.I | | 194 | 124 | N.I | | N.I |  |
| House sparrow 3 | 206 | 125 | | 163 | 72 | | 162 | 109 | 238 | | 125 |  |
| House sparrow 3_Rep | N.I | N.I | | 191 | 90 | | N.I | N.I | 220 | | 97 |  |
| House sparrow 4 | 342 | 188 | | 253 | 146 | | 269 | 192 | 220 | | 149 |  |
| House sparrow 4_Rep | N.I | N.I | | 254 | 151 | | N.I | N.I | 265 | | 159 |  |
| House sparrow 5 | 233 | 116 | | - | - | | 206 | 160 | 500 | | 322 |  |
| Average | 274 | 152 | | 219 | 115 | | 228 | 153 | 265 | | 155 |  |
| Spanish sparrow 1 | 236 | 133 | | 181 | 98 | | 378 | 258 | 450 | | 281 |  |
| Spanish sparrow 1_Rep | 296 | 150 | | N.I | N.I | | 416 | 230 | 418 | | 225 |  |
| Spanish sparrow 2 | 237 | 115 | | 431 | 256 | | 580 | 376 | 435 | | 243 |  |
| Spanish sparrow 2_Rep | 509 | 281 | | N.I | N.I | | 400 | 265 | N.I | | N.I |  |
| Spanish sparrow 3 | 316 | 141 | | 192 | 106 | | 245 | 200 | 282 | | 134 |  |
| Spanish sparrow 3_Rep | N.I | N.I | | N.I | N.I | | N.I | N.I | 331 | | 153 |  |
| Spanish sparrow 4 | 282 | N.I | | 347 | 175 | | N.I | N.I | N.I | | N.I |  |
| Spanish sparrow 4_Rep | N.I | N.I | | 214 | 141 | | N.I | N.I | N.I | | N.I |  |
| Spanish sparrow 5 | 306 | N.I | | 157 | - | | N.I | N.I | N.I | | N.I |  |
| Spanish sparrow 5_Rep | N.I | N.I | | 375 | 215 | | N.I | N.I | N.I | | N.I |  |
| Average | 312 | 164 | | 271 | 165 | | 404 | 266 | 383 | | 207 |  |
| Tree sparrow 1 | 269 | 129 | | - | - | | 300 | 192 | 141 | | - |  |
| Tree sparrow 1_Rep | N.I | N.I | | - | - | | 283 | 192 | N.I | | N.I |  |
| Tree sparrow 2 | 249 | 120 | | 186 | 113 | | 377 | 194 | 313 | | 146 |  |
| Tree sparrow 2_Rep | N.I | N.I | | - | - | | 186 | 111 | N.I | | N.I |  |
| Tree sparrow 3 | 307 | 152 | | 322 | 176 | | 268 | 179 | 239 | | 78 |  |
| Tree sparrow 3_Rep | 210 | 116 | | N.I | N.I | | N.I | N.I | 205 | | 88 |  |
| Tree sparrow 4 | 215 | 106 | | 182 | 117 | | 152 | 97 | 242 | | 93 |  |
| Tree sparrow 4_Rep | 204 | 112 | | N.I | N.I | | N.I | N.I | 148 | | - |  |
| Tree sparrow 5 | 213 | 119 | | 307 | 177 | | 208 | 135 | 217 | | 101 |  |
| Average | 238 | 122 | | 249 | 146 | | 253 | 157 | 215 | | 101 |  |

| (b) | **Primer combination 1** | | **Primer combination 2** | | | **Primer combination 3** | | | | **Primer combination 4** | | |
| --- | --- | --- | --- | --- | --- | --- | --- | --- | --- | --- | --- | --- |
| Individual | Before filtering | After filtering | | Before filtering | After filtering | | Before filtering | After filtering | Before filtering | | After filtering |  |
| House sparrow 1 | 448 | 243 | | 120 | - | | 372 | 267 | 244 | | 167 |  |
| House sparrow 1_Rep | N.I | N.I | | 234 | 149 | | 371 | 204 | N.I | | N.I |  |
| House sparrow 2 | 253 | 162 | | 313 | 162 | | 230 | 168 | 215 | | 141 |  |
| House sparrow 2_Rep | N.I | N.I | | 1 | - | | N.I | N.I | N.I | | N.I |  |
| House sparrow 3 | 299 | 179 | | 623 | 295 | | 86 | - | 302 | | 220 |  |
| House sparrow 3_Rep | 288 | 179 | | N.I | N.I | | 219 | 172 | N.I | | N.I |  |
| House sparrow 4 | 121 | - | | 193 | 126 | | 382 | 255 | 203 | | 147 |  |
| House sparrow 4_Rep | 392 | 235 | | N.I | N.I | | N.I | N.I | 269 | | 178 |  |
| House sparrow 5 | 254 | 187 | | 161 | 91 | | 364 | 268 | 321 | | 203 |  |
| House sparrow 5_Rep | N.I | N.I | | 247 | 156 | | N.I | N.I | 321 | | 208 |  |
| Average | 294 | 198 | | 237 | 163 | | 289 | 222 | 268 | | 181 |  |
| Spanish sparrow 1 | 319 | 189 | | 276 | 174 | | 668 | 482 | 399 | | 263 |  |
| Spanish sparrow 1_Rep | N.I | N.I | | - | - | | 543 | 384 | 585 | | 398 |  |
| Spanish sparrow 2 | 206 | 123 | | 178 | - | | 578 | 410 | 476 | | 277 |  |
| Spanish sparrow 2_Rep | 276 | 152 | | N.I | N.I | | N.I | N.I | 401 | | 237 |  |
| Spanish sparrow 3 | 51 | - | | 375 | 238 | | 614 | 415 | 437 | | 280 |  |
| Spanish sparrow 3_Rep | 89 | - | | N.I | N.I | | 473 | 348 | N.I | | N.I |  |
| Average | 188 | 155 | | 276 | 206 | | 575 | 408 | 460 | | 291 |  |
| Tree sparrow 1 | 146 | - | | 515 | 283 | | 201 | - | 303 | | 226 |  |
| Tree sparrow 1_Rep | N.I | N.I | | N.I | N.I | | N.I | N.I | N.I | | N.I |  |
| Tree sparrow 2 | 470 | 232 | | 265 | 160 | | 329 | 201 | 215 | | 142 |  |
| Tree sparrow 2_Rep | 361 | 202 | | N.I | N.I | | N.I | N.I | 280 | | 174 |  |
| Tree sparrow 3 | 417 | 190 | | 127 | - | | 257 | 162 | 190 | | - |  |
| Tree sparrow 3_Rep | 333 | 165 | | N.I | N.I | | N.I | N.I | 382 | | 275 |  |
| Tree sparrow 4 | 325 | 156 | | 275 | 161 | | 322 | 228 | 240 | | 170 |  |
| Tree sparrow 4_Rep | N.I | N.I | | N.I | N.I | | 267 | 201 | N.I | | N.I |  |
| Tree sparrow 5 | 291 | 161 | | 377 | 227 | | 263 | 162 | 271 | | 198 |  |
| Tree sparrow 5_Rep | N.I | N.I | | 278 | 187 | | 307 | 199 | N.I | | N.I |  |
| Average | 335 | 184 | | 306 | 204 | | 278 | 192 | 269 | | 198 |  |

**Table S4** Comparison of repeatability between samples that were sequenced, in replicates, with 454 amplicon sequencing. First the total number of alleles amplified by each replicate pair was determine as the combined results from the two replicated samples, then the percentage of amplified alleles within each sample was determined. The estimates are reported separately for each species, primer combination and sample type (gDNA/cDNA).The replicated individuals were house sparrow ind 1 and 2, Spanish sparrow ind 1 and 2, tree sparrow ind 3 and 4 (primer combination 1, gDNA), house sparrow ind 3, Spanish sparrow ind 2, tree sparrow ind 2 and 3 (primer combination 1, cDNA), house sparrow ind 3 and 4, Spanish sparrow ind 5 (primer combination 2, gDNA), house sparrow ind 5, tree sparrow ind 5 (primer combination 2, cDNA), house sparrow ind 1 and 2, Spanish sparrow ind 1 and 2, tree sparrow ind 1 and 2 (primer combination 3, gDNA), house sparrow ind 1, Spanish sparrow ind 1 and 3, tree sparrow ind 4 and 5 (primer combination 3, cDNA), house sparrow ind 3 and 4, Spanish sparrow ind 1 and 4, tree sparrow ind 3 (primer combination 4, gDNA) and house sparrow ind 4 and 5, Spanish sparrow ind 1 and 4, tree sparrow ind 2 (primer combination 4, cDNA),

| Species | Sample type | Primer combination 1 | | Primer combination 2 | | Primer combination 3 | | Primer combination 4 | |
| --- | --- | --- | --- | --- | --- | --- | --- | --- | --- |
|  |  | Rep 1 | Rep 2 | Rep 1 | Rep 2 | Rep 1 | Rep 2 | Rep 1 | Rep 2 |
| House sparrow | gDNA | 83% | 83% | 90% | 80% | 100% | 100% | 90% | 80% |
| House sparrow | gDNA | 100% | 100% | 100% | 100% | 100% | 100% | 100% | 100% |
| Spanish sparrow | gDNA | 100% | 77% | 78% | 83% | 100% | 100% | 83% | 100% |
| Spanish sparrow | gDNA | 100% | 70% |  |  | 100% | 100% | 100% | 100% |
| Tree sparrow | gDNA | 100% | 100% | - | - | 100% | 100% | 100% | 100% |
| Tree sparrow | gDNA | 100% | 87.5% | - | - | 100% | 100% |  |  |
|  |  |  |  |  |  |  |  |  |  |
| House sparrow | cDNA | 100% | 86% | 100% | 75% | 100% | 100% | 67% | 100% |
| House sparrow | cDNA | - | - | - | - | - | - | 100% | 100% |
| Spanish sparrow | cDNA | 100% | 83% | - | - | 100% | 100% | 100% | 50% |
| Spanish sparrow | cDNA |  |  | - | - | 100% | 100% | 83% | 83% |
| Tree sparrow | cDNA | 100% | 100% | 100% | 100% | 100% | 100% | 100% | 100% |
| Tree sparrow | cDNA | 71% | 86% | - | - | 100% | 100% | - | - |

**Table S5** Diversity measurements of classical and non-classical MHC-I exon 3 alleles in house sparrow, Spanish sparrow and tree sparrow. The estimates are reported separately for classical (a) and non-classical (b) alleles for all alleles found in the genome (gDNA), for expressed alleles (cDNA) and for alleles that are not expressed.

| (a) |  | Classical alleles | | |
| --- | --- | --- | --- | --- |
| Species | Sample type | Number of nucleotide sequences | Nucleotide diversity | Amino acid seq. per nucleotide seq. ratio |
| House sparrow | gDNA | 18 | 0.110 | 1.00 |
| Spanish sparrow | gDNA | 14 | 0.074 | 0.93 |
| Tree sparrow | gDNA | 24 | 0.086 | 0.96 |
| House sparrow | expressed | 11 | 0.111 | 1.00 |
| Spanish sparrow | expressed | 9 | 0.088 | 0.89 |
| Tree sparrow | expressed | 9 | 0.063 | 1.00 |
| House sparrow | not expressed | 7 | 0.106 | 1.00 |
| Spanish sparrow | not expressed | 5 | 0.021 | 1.00 |
| Tree sparrow | not expressed | 15 | 0.091 | 1.00 |

| (b) |  | Non-classical alleles | | |
| --- | --- | --- | --- | --- |
| Species | Sample type | Number of nucleotide sequences | Nucleotide diversity | Amino acid seq. per nucleotide seq. ratio |
| House sparrow | gDNA | 30 | 0.023 | 0.7 |
| Spanish sparrow | gDNA | 30 | 0.027 | 0.8 |
| Tree sparrow | gDNA | 13 | 0.018 | 0.92 |
| House sparrow | expressed | 16 | 0.023 | 0.75 |
| Spanish sparrow | expressed | 16 | 0.027 | 0.75 |
| Tree sparrow | expressed | 5 | 0.016 | 0.8 |
| House sparrow | not expressed | 14 | 0.022 | 0.86 |
| Spanish sparrow | not expressed | 14 | 0.028 | 0.93 |
| Tree sparrow | not expressed | 8 | 0.019 | 1.00 |

**Table S6** The number of putatively classical and non-classical MHC-I alleles found in the genome (gDNA) as well as number of expressed alleles (cDNA) in house sparrows, Spanish sparrows and tree sparrows. All individuals were sequenced with four different primer combinations and the consensus result where used for determining the number of alleles in each individual. Letters indicate significant differences (p<0.05), comparisons are made within sample type and gene type.

|  | **Classical** | | **Non-classical** | |
| --- | --- | --- | --- | --- |
|  | gDNA | cDNA | gDNA | cDNA |
| House sparrow individual 1 | 7 | 4 | 13 | 4 |
| House sparrow individual 2 | 5 | 3 | 6 | 4 |
| House sparrow individual 3 | 5 | 3 | 10 | 4 |
| House sparrow individual 4 | 3 | 2 | 5 | 3 |
| House sparrow individual 5 | 1 | 1 | 7 | 5 |
| **Average House sparrows** | 4±2 ^a^ | 3±1 ^c^ | 8±3 ^d e^ | 4±1 ^f^ |
| Spanish sparrow individual 1 | 6 | 3 | 12 | 6 |
| Spanish sparrow individual 2 | 5 | 4 | 12 | 4 |
| Spanish sparrow individual 3 | 5 | 2 | 9 | 6 |
| **Average Spanish sparrows** | 5±1 ^a^ | 3±1 ^c^ | 11±2 ^d^ | 5±1 ^f^ |
| Tree sparrow individual 1 | 9 | - | 3 | 1 |
| Tree sparrow individual 2 | 14 | 4 | 7 | 3 |
| Tree sparrow individual 3 | 9 | 4 | 4 | 3 |
| Tree sparrow individual 4 | 8 | 3 | 5 | 1 |
| Tree sparrow individual 5 | 8 | 3 | 4 | 2 |
| **Average Tree sparrows** | 10±3 ^b^ | 4±1 ^c^ | 5±2 ^e^ | 2±1 ^g^ |

**Table S7** The putatively classical and non-classical MHC-I alleles identified in house sparrows, Spanish sparrows and tree sparrows. The star (*) indicates expressed alleles and marked in bold and underlined are the alleles that were identified as the most highly expressed, marked both for classical and non classical, during the expression analysis performed on three house sparrows (house sparrow individual 2, 3, 4) and three tree sparrows (tree sparrow individual 2, 3, 4).

| House sparrow individual 1 | House sparrow individual 2 | House sparrow individual 3 | House sparrow individual 4 | House sparrow individual 5 |
| --- | --- | --- | --- | --- |
| Pado-UA_245a | Pado-UA_239a* | Pado-UA_359* | **Pado-UA_258a*** | Pado-UA_319a* |
| Pado-UA_248a* | Pado-UA_256a | Pado-UA_238a | Pado-UA_297a* |  |
| Pado-UA_278a* | Pado-UA_257a | Pado-UA_256a | Pado-UA_358 | Pado-UA*204a* |
| Pado-UA_315a | **Pado-UA_361*** | Pado-UA_361* |  | Pado-UA*205a |
| Pado-UA_357 | Pado-UA_362* | **Pado-UA_362*** | **Pado-UA*202a*** | Pado-UA*226a |
| Pado-UA_360* |  |  | Pado-UA*220a* | Pado-UA*309a* |
| Pado-UA_363* | Pado-UA*204a* | Pado-UA*202a* | Pado-UA*223a* | Pado-UA*365* |
|  | Pado-UA*219a | Pado-UA*218a | Pado-UA*309a | Pado-UA*367* |
| Pado-UA*201a | Pado-UA*303a* | Pado-UA*219a | Pado-UA*368 | Pado-UA*371* |
| Pado-UA*206 | Pado-UA*308a* | Pado-UA*226a |  |  |
| Pado-UA*209a* | Pado-UA*364 | Pado-UA*227a |  |  |
| Pado-UA*225a* | **Pado-UA*370*** | **Pado-UA*309a*** |  |  |
| Pado-UA*227a |  | Pado-UA*324a* |  |  |
| Pado-UA*310a |  | Pado-UA*364 |  |  |
| Pado-UA*366 |  | Pado-UA*370* |  |  |
| Pado-UA*369 |  | Pado-UA*371 |  |  |
| Pado-UA*371 |  |  |  |  |
| Pado-UA*372 |  |  |  |  |
| Pado-UA*373* |  |  |  |  |
| Pado-UA*374* |  |  |  |  |
| Pado-UA*375 |  |  |  |  |

| Spanish sparrow individual 1 | Spanish sparrow individual 2 | Spanish sparrow individual 3 |  |  |
| --- | --- | --- | --- | --- |
| Pahi-UA*31a | Pahi-UA*31a* | Pahi-UA*41* |  |  |
| Pahi-UA*32a* | Pahi-UA*34a* | Pahi-UA*46* |  |  |
| Pahi-UA*36 | Pahi-UA*36* | Pahi-UA*47 |  |  |
| Pahi-UA*40* | Pahi-UA*42* | Pahi-UA*48 |  |  |
| Pahi-UA*43* | Pahi-UA*45 | Pahi-UA*49 |  |  |
| Pahi-UA*44 | Pahi-UA*08a |  |  |  |
|  | Pahi-UA*14a | Pahi-UA*02a* |  |  |
| Pahi-UA*04* | Pahi-UA*19 | Pahi-UA*16* |  |  |
| Pahi-UA*10* | Pahi-UA*22 | Pahi-UA*19 |  |  |
| Pahi-UA*19 |  | Pahi-UA*21* |  |  |
| Pahi-UA*20* | Pahi-UA*27* | Pahi-UA*51* |  |  |
| Pahi-UA*26* | Pahi-UA*53 | Pahi-UA*53 |  |  |
| Pahi-UA*28a | Pahi-UA*55 | Pahi-UA*57* |  |  |
| Pahi-UA*50* | Pahi-UA*56* | Pahi-UA*60 |  |  |
| Pahi-UA*52 | Pahi-UA*59 | Pahi-UA*62* |  |  |
| Pahi-UA*54 | Pahi-UA*61 |  |  |  |
| Pahi-UA*58 | Pahi-UA*63* |  |  |  |
| Pahi-UA*64 | Pahi-UA*65* |  |  |  |
| Pahi-UA*100* |  |  |  |  |
| Tree sparrow Individual 1 | Tree sparrow Individual 2 | Tree sparrow Individual 3 | Tree sparrow Individual 4 | Tree sparrow Individual 5 |
| Pamo-UA*12a | Pamo-UA*13a | **Pamo-UA*20a*** | **Pamo-UA*31*** | Pamo-UA*12a |
| Pamo-UA*13a | Pamo-UA*14a | Pamo-UA*13a | Pamo-UA*14a | Pamo-UA*13a |
| Pamo-UA*14a | Pamo-UA*18a | Pamo-UA*16a | Pamo-UA*16a | Pamo-UA*18a |
| Pamo-UA*17a | Pamo-UA*19a | Pamo-UA*17a* | Pamo-UA*20a* | Pamo-UA*19a |
| Pamo-UA*18a | **Pamo-UA*20a*** | Pamo-UA*18a | Pamo-UA*23a | Pamo-UA*22a |
| Pamo-UA*27a | Pamo-UA*22a | Pamo-UA*22a | Pamo-UA*26a | Pamo-UA*25a* |
| Pamo-UA*28 | Pamo-UA*23a | Pamo-UA*26a | Pamo-UA*27a* | Pamo-UA*29* |
| Pamo-UA*33 | Pamo-UA*25a* | Pamo-UA*27a* | Pamo-UA*33 | Pamo-UA*35* |
| Pamo-UA*36 | Pamo-UA*26a | Pamo-UA*37* |  |  |
|  | Pamo-UA*27a |  | **Pamo-UA*02a*** | Pamo-UA*02a* |
| Pamo-UA*02a* | Pamo-UA*30* | **Pamo-UA*02a*** | Pamo-UA*05a | Pamo-UA*07a |
| Pamo-UA*04a | Pamo-UA*32 | Pamo-UA*05a | Pamo-UA*09a | Pamo-UA*09a* |
| Pamo-UA*06a | Pamo-UA*34 | Pamo-UA*06a* | Pamo-UA*10a | Pamo-UA*43 |
|  | Pamo-UA*38* | Pamo-UA*39* | Pamo-UA*40 |  |
|  |  |  |  |  |
|  | **Pamo-UA*02a*** |  |  |  |
|  | Pamo-UA*03a |  |  |  |
|  | Pamo-UA*04a |  |  |  |
|  | Pamo-UA*09a* |  |  |  |
|  | Pamo-UA*39 |  |  |  |
|  | Pamo-UA*41* |  |  |  |
|  | Pamo-UA*42 |  |  |  |

**Table S8** Comparison of relative read depth per allele between two of the three tree sparrow individuals that were used for the expression analysis.

| **Individual** | **Type of gene** | **Allele** | **Relative read depth** | |
| --- | --- | --- | --- | --- |
| Tree sparrow ind 2 | Classical | Pamo-UA*20a | 0.43 | 0.46 |
| Tree sparrow ind 2 | Classical | Pamo-UA*30 | 0.34 | 0.37 |
| Tree sparrow ind 2 | Classical | Pamo-UA*25a | 0.01 | 0.004 |
| Tree sparrow ind 2 | Classical | Pamo-UA*38 | 0.11 | 0.09 |
| Tree sparrow ind 2 | Non-classical | Pamo-UA*02a | 0.08 | 0.06 |
| Tree sparrow ind 2 | Non-classical | Pamo-UA*09a | 0.01 | 0.004 |
| Tree sparrow ind 2 | Non-classical | Pamo-UA*41 | 0.02 | 0.02 |
|  |  |  |  |  |
| Tree sparrow ind 3 | Classical | Pamo-UA*17a | 0.31 | 0.32 |
| Tree sparrow ind 4 | Classical | Pamo-UA*20a | 0.55 | 0.58 |
| Tree sparrow ind 5 | Classical | Pamo-UA*37 | 0.03 | 0.00 |
| Tree sparrow ind 6 | Classical | Pamo-UA*27a | 0.00 | 0.01 |
| Tree sparrow ind 7 | Non-classical | Pamo-UA*02a | 0.11 | 0.08 |
| Tree sparrow ind 8 | Non-classical | Pamo-UA*06a | 0.00 | 0.01 |
| Tree sparrow ind 9 | Non-classical | Pamo-UA*39 | 0.01 | 0.01 |

**Table S9** Six individuals (three house sparrows and three tree sparrows) individuals where primer combination 1 amplifications satisfactory picked up putatively classical and non-classical alleles were selected for the expression analysis. Close to complete amplification of all expressed alleles (two tree sparrow individuals (4 and 2) are both missing one low expression classical allele) where obtained with primer combination 1. Primer combination 1 amplifies a shorter fragment than the other primer combinations meaning that not all alleles could not be distinguished with this combination. However they could be distinguished with one of the other primer combinations. In these cases read depth obtained for primer combination 1 was split according to the two alleles proportions when amplified with one of the other combinations (indicated by ^*^).

| Individual | Classical | Non-classical |
| --- | --- | --- |
| House Sparrow 2 | 3 out of 3 | 4 out of 4 |
| House Sparrow 3 | 3 out of 3 | 4 out of 4 |
| House Sparrow 4 | 2 out of 2 | 3 out of 3 |
| Tree sparrow 2 | 4^*^ out of 4 | 3^*^out of 3 |
| Tree sparrow 3 | 3 out of 4 | 3 out of 3 |
| Tree sparrow 4 | 2 out of 3 | 1 out of 1 |

1

3 & 4

2

2, 3 & 4

1

Exon 3

**Fig. S1** Schematic overview of MHC exon 3 where the arrows indicates the different locations of the primers used in this study. Primer combination 1 (HNalla-RVS3B) amplifies a 185-191 bp long fragment, Primer combination 2 (FWD3-RVS) amplifies a 220-226 bp long fragment, Primer combination 3 (Long-RVS) amplifies a 229-232 bp long fragment and Primer combination 4 (Short-RVS) amplifies a 226 bp long fragment.

**Fig. S2** Amino acid alignment of house sparrow (Pado), Spanish sparrow (Pahi) and tree sparrow (Pamo) MHC-I exon 3 alleles where identity to Pado-UA*238a is indicated by dots. Alignment contains two groups, putatively classical alleles (Pado-UA*238a-239a, Pado-UA*245a, Pado-UA*248a, Pado-UA*256, Pado-UA*258a, Pado-UA*278a, Pado-UA*297a, Pado-UA*315a, Pado-UA*319a, Pado-UA*357-363; Pahi-UA*34a, Pahi-UA*36, Pahi-UA*40-49; Pamo-UA*12a-14a, Pamo-UA*16a-20a, Pamo-UA*22a-23a, Pamo-UA*25a-38) and putatively non-classical alleles (Pado-UA*201a-202a, Pado-UA*204a-206, Pado-UA*209a, Pado-UA*218a-220a, Pado-UA*223a, Pado-UA*22a5-227a, Pado-UA*303a, Pado-UA*308a-310a, Pado-UA*324a, Pado-UA*364-375; Pahi-UA* 2a, Pahi-UA*4, Pahi-UA*8a, Pahi-UA*10, Pahi-UA*14a, Pahi-UA*16, Pahi-UA*19-22, Pahi-UA*26-28a, Pahi-UA*50-65, Pahi-UA*100; Pamo-UA*2a-10, Pamo-UA*39-41). In non-classical alleles two amino acids have been deleted, see position 54-55. There are two motifs that are unique to the putatively non-classical alleles, 51 H and 80 C, these are indicated with boxes. The H in position 51 was also identified by Karlsson and Westerdahl (2013) as unique for non-classical alleles.





**Fig. S3** Neighbor-net network based on MHC-I alleles (exon 3 sequences) from house sparrows (Pado; indicated in green), Spanish sparrows (Pahi; indicated in orange) and tree sparrows (Pamo; indicated in purple) amplified with primer combination 1. Stars (*) indicates alleles that were found in both gDNA and cDNA (*i.e.* expressed alleles) and the bootstrap percentage value display the support for the split between putatively classical and non-classical alleles (bootstrap=93.4). All putatively non-classical alleles are found in the left cluster, with no clustering based on species, whereas the putatively classical alleles do not form a distinct cluster.


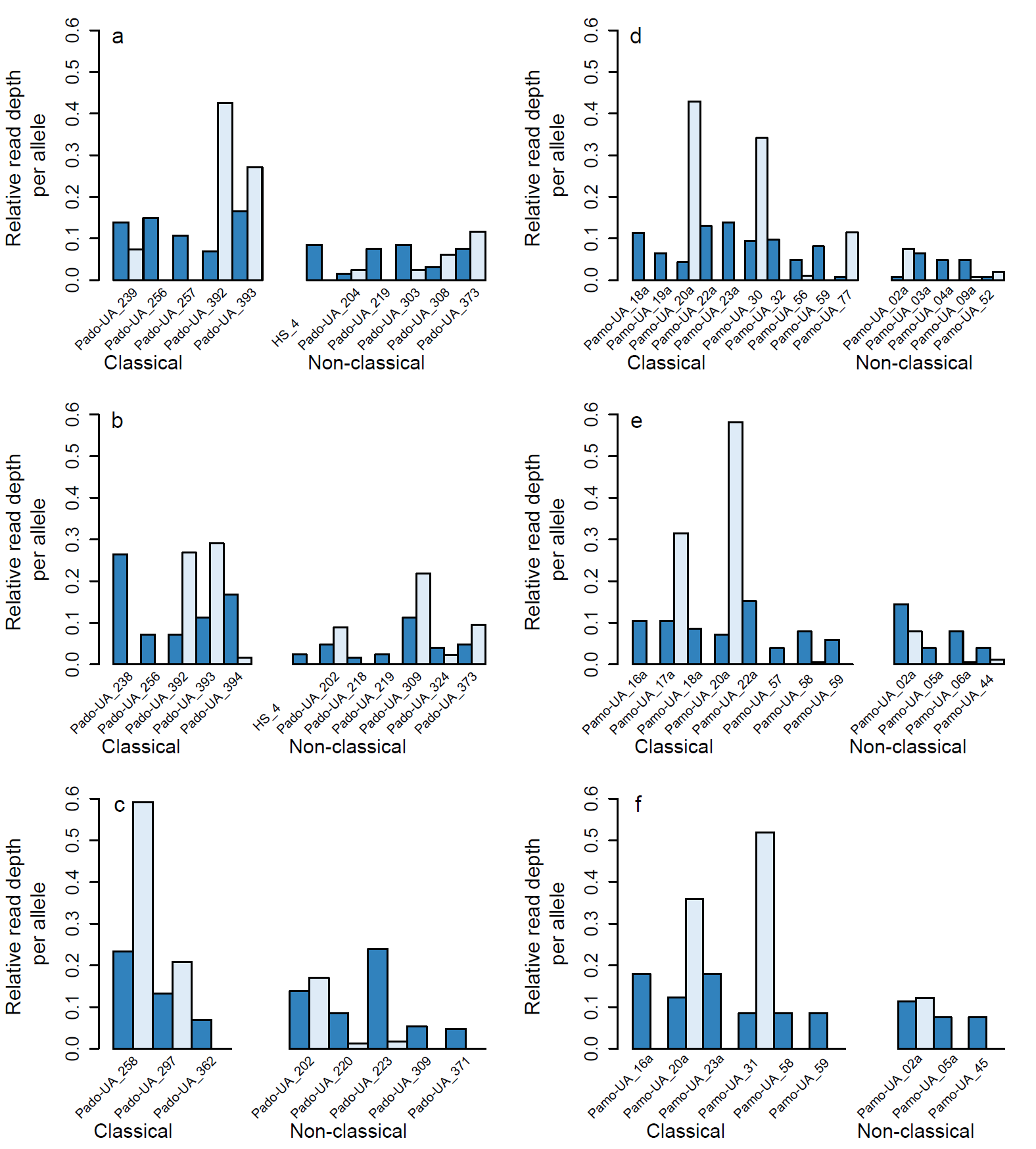


**Fig. S4** The proportion of reads per allele in the six selected individuals for the expression analysis. a = house sparrow 2, b = house sparrow 3, c = house sparrow 4, d = tree sparrow 2, e = tree sparrow 3, f = tree sparrow 4. On the x-axis are all alleles identified in each individual and proportions of reads per allele are given for alleles amplified by primer combination 1. Proportion of reads that an allele has in gDNA (black) and, if the allele is expressed, the proportion of reads in cDNA (grey).

**References**

1. Jetz W, Thomas GH, Joy JB, Hartmann K, Mooers AO. The global diversity of birds in space and time. Nature. 2012;491:444–8.

2. Hackett SJ, Kimball RT, Reddy S, Bowie RCK, Braun EL, Braun MJ, et al. A phylogenomic study of birds reveals their evolutionary history. Science. 2008;320:1763–8.

3. Drummond AJ, Suchard MA, Xie D, Rambaut A. Bayesian phylogenetics with BEAUti and the BEAST 1.7. Mol. Biol. Evol. 2012;29:1969–73.

4. Stuglik MT, Radwan J, Babik W. jMHC: Software assistant for multilocus genotyping of gene families using next-generation amplicon sequencing. Mol. Ecol. Resour. 2011;11:739–42.

5. Huse SM, Huber J a, Morrison HG, Sogin ML, Welch DM. Accuracy and quality of massively parallel DNA pyrosequencing. Genome Biol. 2007;8:R143.

6. Hall T. BioEdit: a user-friendly biological sequence alignment editor and analysis program for Windows 95/98/NT. Nucleic Acids Symp. Ser. 1999. p. 95–8.

7. Galan M, Guivier E, Caraux G, Charbonnel N, Cosson J-F. A 454 multiplex sequencing method for rapid and reliable genotyping of highly polymorphic genes in large-scale studies. BMC Genomics. 2010;11:296.

8. Westerdahl H, Wittzell H, Schantz T von, Bensch S. MHC class I typing in a songbird with numerous loci and high polymorphism using motif-specific PCR and DGGE. Heredity (Edinb). 2004;92:534–42.

9. O’Connor EA, Strandh M, Hasselquist D, Nilsson J, Westerdahl H. The evolution of highly variable immunity genes across a passerine bird radiation. Mol. Ecol. 2016;25:977–89.

10. Karlsson M, Westerdahl H. Characteristics of MHC Class I Genes in House Sparrows Passer domesticus as Revealed by Long cDNA Transcripts and Amplicon Sequencing. J. Mol. Evol. 2013;77:8–21.
